# Analysis of healthcare-associated infection in patients with pulmonary arterial hypertension associated with congenital heart disease in PICU: Evidence from a tertiary hospital in western China

**DOI:** 10.3389/fped.2022.1076618

**Published:** 2022-12-21

**Authors:** Jing-wen Li, Ling-wen Guo, Si-yuan Tao, Yu-hua Deng, Cui Yang, Fu Qiao

**Affiliations:** ^1^ Department of Infection Control, West China Hospital, Sichuan University, Chengdu, China; ^2^ Department of Respiratory and Critical Care Medicine, West China Hospital, Sichuan University, Chengdu, China

**Keywords:** healthcare-associated infection, pediatric intensive care unit, device utilization rate, targeted surveillance, pulmonary arterial hypertension associated with congenital heart disease, device-related infection

## Abstract

**Objective:**

The present study intends to analyze the targeted surveillance and risk factors for healthcare-associated infection (HAI) in patients with pulmonary arterial hypertension associated with congenital heart disease (CHD-PAH) in a Pediatric intensive care unit (PICU), and provide basis for formulating relevant prevention and control measures of HAI.

**Methods:**

Children (≤14 years old) who were admitted to the PICU for ≥2 calendar days from January 2018 to December 2021 were included. Targeted surveillance of HAI was described.

**Results:**

A total of 7,828 patients in PICU were monitored, and the total hospitalization days of the patients were 36,174. 108 cases of HAI occurred, with a per-case infection rate of 1.38% and a per-thousand day infection rate of 2.99. 1,129 patients with CHD-PAH were included, among which the total hospitalization days were 1,483. In this subpopulation, 38 cases of HAI were diagnosed, with a per-case infection rate of 3.37% and a per-thousand day infection rate of 25.62. The main site of HAI was lower respiratory tract (43.51%), followed by blood infection (34.26%) and surgical site infection (9.26%). 36 strains of pathogenic bacteria were detected from patients with HAI. The top three pathogens with the highest detection rate were Klebsiella pneumoniae (6 episodes, 16.67%), Enterococcus faecium (6 episodes, 16.67%) and Acinetobacter baumannii (4 episodes, 11.11%). The incidence of VAP, CAUTI and CLABSI was 2.78, 0.08 and 1.66 per 1,000 catheter days respectively. Analysis revealed that patients with CHD-PAH were younger and prone to receive surgical corrections. CHD-PAH could significantly increase the length of ICU stay, ventilator days, times of central venous catheterization and central venous catheterization days. The choice of different central venous catheter types differed significantly between the two groups.

**Conclusion:**

Patients with CHD-PAH are characterized with excessive central venous catheterization operations, prolonged indwelling time, and more types of catheterization, which are considered to be risk factors for HAI, thus increasing the length of hospital stay. The clinical etiology is mainly G-bacteria, which requires reasonable selection of antibiotics and strict aseptic operation. Limiting unnecessary invasive procedures is helpful for reducing the incidence of postoperative HAI in PICU.

## Introduction

Healthcare-associated infection (HAI) impacts care and costs, and is one of significant causes of morbidity and mortality while in hospital. The pediatric intensive care unit (PICU) is the department treating high-risk pediatric patients in the hospital. A variety of causes could contribute to the increased risk of HAI in PICU, including the intrinsic factors such as young age, low nutritional status, immature immune system, and extrinsic factors such as the presence of multi-resistant bacteria in the environment, numerous invasive operations and so on (1). A recent study in developing country suggested that there were 27.5 and 33.0 HAIs per 1,000 patient days in PICU, which was significantly higher than that in the non-ICU setting, with 6.8 HAIs per 1,000 patient days in both 2013 and 2014 (2). HAI is serious and important in PICU, and effective surveillance will allow the development and evaluation of targeted interventions to improve care of patients.

Congenital heart disease (CHD) is one of the most common birth defects, which seriously threatens the health and life of patients. Long term left-to-right shunt in the heart will result in increased pulmonary blood flow or pulmonary vascular resistance, leading to pulmonary arterial hypertension (PAH). World symposium on Pulmonary Hypertension classified CHD-PAH into the first category (3), which has been proven to be closely related to the impairment in functional class, quality of life, and survival. It was reported that the overall cumulative incidence of PAH in CHD was 5%–10% (4), and the 1, 5 and 10 year mortality in CHD-PAH was 24%, 44% and 52% respectively (5). As well known, most congenital heart diseases require surgical correction. Cardiac surgery, especially cardiopulmonary bypass, could worsen damage to the lungs and other systems, making patients more susceptible to infections. Previous literature has found that nearly 40.0% CHD children in hospital were tested as positive by sputum culture, predominantly induced by Gram-positive bacteria susceptible to cephalosporins and vancomycin (6). However, there are few studies on various types and risk of HAI in patients with CHD-PAH.

In the present study, we intend to analyze clinical characteristics of all patients in a single PICU from a tertiary general hospital in China, and provide a detailed description of HAI in children with CHD-PAH.

## Materials and methods

### Study settings

This study was conducted in a tertiary general hospital in western China. Consecutive pediatric patients who were admitted to the PICU from January 2018 to December 2021 were included. Inclusion criteria: (1) age ≤14 years old; (2) patients receiving treatment in PICU for ≥2 calendar days. Exclusion criteria: (1) patients with infections before transferred into PICU; (2) patients who died within 48 h; (3) patients with incomplete clinical data. All included patients were follow-up for 2 calendar days after transferring out of PICU. CHD was diagnosed based on echocardiography, and PAH was diagnosed by tricuspid valve regurgitation pressure method (7).

### Surveillance process

According to the Norms of nosocomial Infection Surveillance issued by the National Health Commission of the People's Republic of China in 2009, a targeted surveillance program was formulated, and the monitoring data were collected by the professional HAI staff. The nurses in PICU evaluated the admitted patients daily and filled in the Registration Form of ICU Patients’ Targeted Monitoring Survey and ICU Patient Log. The survey included the indwelling situation of ventilators, central venous catheters, urinary catheters and other instruments and the detection of pathogenic microorganisms. The contents of Log included daily number of new patients admitted in the PICU, daily number of patients out of the PICU, daily number of patients using invasive devices, etc. The clinicians in PICU evaluated the severity of the disease at fixed time every day. The HAI staff monitored and reported HAI cases in real time through the electronic system. By summarizing and analyzing Logs of patients, information about HAI cases, pathogenic bacteria detection, usage of invasive devices and related infections was fed back to the PICU, in order to facilitate continuous quality improvement, prevention and control of potential infection risk.

### Definitions

HAI referred to new onset infections occurring after hospital admission for more than 48 h. Various types of HAI were defined based on the criteria established by the Chinese Ministry of Health (8), including ventilator associated pneumonia (VAP), catheter associated urinary tract infection (CAUTI), central line associated bloodstream infection (CLABSI), and so on. If one patient was diagnosed with more than one type of HAI, they were counted in each category. Clinical data were collected through electronic medical record system.

### Data analysis

Data were analyzed by SPSS 25.0 software. Measurement data were expressed as *n* (percent), and comparisons between groups were performed by using chi-square test. Per-thousand day infection rate between groups were compared by using Poisson test. *P* ≤ 0.05 was considered to be statistically significant.

## Results

### Incidence of HAI in PICU

As Tables 1, 2 showed, from January 2018 to December 2021, a total of 7,828 patients in PICU were monitored, and the total hospitalization days of the patients were 36,174. 108 cases of HAI occurred, with a per-case infection rate of 1.38% and a per-thousand day infection rate of 2.99. 1,129 patients with CHD-PAH were included, among which the total hospitalization days were 1,483 days. In this subpopulation, 38 cases of HAI were diagnosed, with a per-case infection rate of 3.37% and a per-thousand day infection rate of 25.62. Compared to patients without CHD-PAH, both the per-case infection rate and per-thousand day infection rate in patients with CHD-PAH significantly increased in the year of 2018 and 2019. The presence of CHD-PAH could also increase the overall incidence of HAI from 2018 to 2021.

**Table 1:** Summary of per-case infection rate of HAI.

Year	Total patients			CHD-PAH patients			*χ* ^2^	*P*
	Number of patients	Infection cases	Per-case infection rate (%)	Number of patients	Infection cases	Per-case infection rate (%)		
2018	2,041	41	2.01	435	17	3.91	10.130*	**0.001** *
2019	2,032	26	1.28	203	13	6.40	46.885*	**<0.001** *
2020	1,885	19	1.01	302	4	1.32	0.082*	0.774*
2021	1,870	22	1.18	189	4	2.12	0.825*	0.363*
	7,828	108	1.38	1,129	38	3.37	38.249*	**<0.001** *

Bold value indicates the difference was significant.

* Comparisons between CHD-PAH patients and non-CHD-PAH patients.

**Table 2:** Summary of per-thousand day infection rate of HAI.

Year	Total patients			CHD-PAH patients			*U*	*P*
	Hospitalization days	Infection cases	Per-thousand day infection rate (‰)	Hospitalization days	Infection cases	Per-thousand day infection rate (‰)		
2018	9,241	41	4.44	489	17	34.76	3.790*	**<0** **.** **001** *
2019	9,061	26	2.87	617	13	21.07	3.333*	**0** **.** **001** *
2020	8,719	19	2.18	131	4	30.53	1.885*	0.059*
2021	9,153	22	2.40	246	4	16.26	1.748*	0.080*
	36,174	108	2.99	1,483	38	25.62	5.669*	**<0** **.** **001** *

Bold value indicates the difference was significant.

* Comparisons between CHD-PAH patients and non-CHD-PAH patients.

### Site distribution of HAI

As Table 3 showed, the main site of HAI was lower respiratory tract (43.51%), followed by blood infection (34.26%) and surgical site infection (9.26%). Abdominal pelvic (5.56%), skin (4.63%) and urinary (2.78%) were the three areas with the lowest incidence of HAI.

**Table 3:** Distribution of HAI sites in patients in PICU.

Sites	Infection cases	*n* (%)
Lower respiratory tract	47	43.51
Blood	37	34.26
Surgical site infection	10	9.26
Abdominal pelvic	6	5.56
Skin	5	4.63
Urinary	3	2.78
Total	108	100

### Etiological distribution of HAI

After excluding duplicate strains isolated from the same site of the same patient, a total of 36 strains of pathogenic bacteria were detected from patients with HAI, including Gram-negative bacteria (21 episodes, 58.33%), Gram-positive bacteria (11 episodes, 30.56%) and fungi (4 episodes, 11.11%). The top three pathogens with the highest detection rate were Klebsiella pneumoniae (6 episodes, 16.67%), Enterococcus faecium (6 episodes, 16.67%) and Acinetobacter baumannii (4 episodes, 11.11%).

### Invasive device-related infections

From 2018 to 2021, the overall number of ventilator days was 18,012, the rate of ventilator use was 49.79%, and the incidence of VAP per 1,000 ventilator days was 2.78. The overall number of urinary catheterization days was 24,437, the rate of urinary catheter use was 67.55%, and the incidence of CAUTI per 1,000 catheter days was 0.08. The overall number of central venous catheterization days was 25,371, the rate of central venous catheterization use was 70.14%, and the incidence of CLABSI per 1,000 catheter days was 1.66. The proportion of device-related infection in HAI was 46.29%, 1.85%, and 38.89% respectively (Table 4).

**Table 4:** Summary of invasive device-related infections in PICU.

Year	Total Hospitalization days (days)	Ventilator				Urinary catheter				Central venous catheter			
		Usage days (days)	Usage rate (%)	Infection cases	VAP incidence per 1,000 ventilator days	Usage days (days)	Usage rate (%)	Infection cases	CAUTI incidence per 1,000 catheters days	Usage days (days)	Usage rate (%)	Infection cases	CLABSI incidence per 1,000 catheters days
2018	9,241	3,949	42.73	15	3.80	5,108	55.28	1	0.20	5,914	64.00	14	2.37
2019	9,061	4,253	46.94	17	4.00	5,882	64.92	0	0	6,093	67.24	12	1.97
2020	8,719	4,105	47.08	11	2.68	6,213	71.26	0	0	6,137	70.39	6	0.98
2021	9,153	5,705	62.33	7	1.23	7,234	79.03	1	0.14	7,227	78.96	10	1.38
	36,174	18,012	49.79	50	2.78	24,437	67.55	2	0.08	25,371	70.14	42	1.66

### Comparisons between patients with or without CHD-PAH

As Table 5 showed, the patients were younger in CHD-PAH group (*χ*^2^ = 16.649, *P* = 0.001). CHD-PAH could significantly increase the length of ICU stay (*χ*^2^ = 6.255, *P* = 0.012), ventilator days (*χ*^2^ = 7.768, *P* = 0.021), times of central venous catheterization (*χ*^2^ = 10.824, *P* = 0.004) and central venous catheterization days (*χ*^2^ = 8.563, *P* = 0.036). The choice of different central venous catheter types differed significantly between the two groups (*χ*^2^ = 10.824, *P* = 0.004). Patients with CHD-PAH were prone to receive surgical corrections (*χ*^2^ = 8.407, *P* = 0.004).

**Table 5:** Comparisons of clinical characteristics between patients with or without CHD-PAH.

	Total	CHD-PAH		*χ* ^2^	*P*
		YES	NO		
Total	108	38 (35.2)	70 (64.8)		
Age				16.649	**0** **.** **001**
Newborn (0–28 days)	39	21 (55.3)	18 (25.7)		
Infant (28 days–1 year)	36	13 (34.2)	23 (32.9)		
Young children (2–3 years)	8	3 (7.9)	5 (7.1)		
Children (4–14 years)	25	1 (2.6)	24 (34.3)		
Gender				0.002	0.964
Male	60	21 (55.3)	39 (55.7)		
Female	48	17 (44.7)	31 (44.3)		
HAI types				6.250	0.619
VAP	42	17 (44.7)	25 (35.7)		
CAUTI	2	1 (2.6)	1 (1.4)		
CLABSI	30	10 (26.3)	20 (28.6)		
Non-VAP	5	2 (5.3)	3 (4.3)		
Non-CAUTI	1	0	1 (1.4)		
Non-CLABSI	7	4 (10.5)	3 (4.3)		
SSI	10	3 (7.9)	7 (10.0)		
Others	11	1 (2.6)	10 (14.3)		
Length of ICU stay (days)				6.255	**0** **.** **012**
≤14	10	0	10 (17.9)		
>14	77	31 (100)	46 (82.1)		
Types of endotracheal intubation				5.983	0.051
NO	8	0	8 (11.4)		
Oral intubation	98	38 (100)	60 (85.7)		
Tracheotomy	2	0	2 (2.9)		
Times of tracheal intubation				11.080	0.110
0	10	0	10 (14.7)		
1	68	29 (76.3)	39 (57.4)		
2	22	5 (13.2)	17 (25.0)		
3	6	4 (10.5)	2 (2.9)		
Ventilator days (days)				7.768	**0** **.** **021**
0	12	1 (2.6)	11 (15.7)		
≤14	37	10 (26.3)	27 (38.6)		
>14	59	27 (71.1)	32 (45.7)		
Types of Central venous catheterization				10.824	**0** **.** **004**
PICC	19	10 (26.3)	9 (13.4)		
CVC	31	4 (10.5)	27 (40.3)		
PICC + CVC	55	24 (63.2)	31 (46.3)		
Times of Central venous catheterization				8.563	**0** **.** **036**
0	3	0	3 (4.3)		
1	48	11 (28.9)	37 (52.9)		
2	43	20 (52.6)	23 (32.9)		
3	14	7 (18.4)	7 (10.0)		
Central venous catheterization days (days)				6.300	**0** **.** **043**
0	5	0	5 (7.1)		
≤14	27	6 (15.8)	21 (30.0)		
>14	76	32 (84.2)	44 (62.9)		
Times of Urinary catheter				3.821	0.281
0	6	0	6 (8.6)		
1	75	27 (71.1)	48 (68.6)		
2	23	9 (23.7)	14 (20.0)		
3	4	2 (5.3)	2 (2.9)		
Urinary catheter days (days)				4.515	0.105
0	7	0	7 (10.0)		
≤14	36	12 (31.6)	24 (34.3)		
>14	65	26 (68.4)	39 (55.7)		
Use of antibiotics				0.530	0.467
0	103	37 (97.4)	66 (94.3)		
1	5	1 (2.6)	4 (5.7)		
Surgery				8.407	**0** **.** **004**
0	12	0	12 (21.4)		
1	78	34 (100)	4478.6 ()		

Bold value indicates the difference was significant.

## Discussion

Compared to adult patients, organs in pediatric patients are not fully developed. Immature immune system, couple with underlying diseases, could lead to weak resistance to external pathogens. Hospital and even ICU setting is a place where various pathogens accumulate (9, 10). In addition with a mass of invasive operations such as puncture and intubation, pediatric patients are susceptible to HAI. The results showed that per-case infection rate of HAI in PICU from 2018 to 2021 was 1.38%, and the per-thousand-day infection rate was 2.99. Previous studies reported the incidence of HAI was almost over ten percent in developing countries (1, 11–13). This significant progress attributed mostly to the thorough and comprehensive surveillance management of HAI in our single center. The three-level management mode was adopted for HAI prevention and control, including hospital's HAI management committee, professional department of HAI, and side-bar HAI team in clinical department. Each patient follows a different specific regimen for HAI, executed by professional HAI staff, nurses and clinicians together.

The proportion of device-related infections was not consistent across hospitals. Hatachi and his colleagues found that CAUTI was the leading one (14), while another study in Turkey reported that the most commonly observed HAI was bloodstream infection (15). Different from above two researches, we found that the main site of nosocomial infection was lower respiratory tract, and VAP was the most likely to appear, accounting for 46.29%. It was probably because that most patients in our PICU were postoperative, who needed the support of mechanical ventilation. Similarly, pathogens will change greatly because of different hospitals in different regions. Our study reported that Klebsiella pneumoniae and Enterococcus faecium were the most frequently isolated microorganism, which provided a basis for the selection of antibiotics in early period.

Our study was the first one focusing on HAI in CHD-PAH. We found that the per-thousand-day infection rate of HAI in patients CHD-PAH was significantly higher than that in patients without CHD-PAH. PAH is a fatal complication of CHD, characterized by progressively increased pulmonary vascular resistance and pulmonary artery pressure (16). Excessive pulmonary congestion makes the lungs more susceptible to bacterial attack (17). Surgery is the main treatment method for children with CHD. After undergoing cardiopulmonary bypass, systemic immune system could further deteriorate (18). All these factors determine higher incidence of HAI in patients with CHD-PAH. On the other hand, local inflammatory reaction in the lung could induce spasm and contraction of pulmonary arterioles, and then cause the increase of pulmonary circulation resistance, aggravating pulmonary hypertension (19). Therefore, it's usually thought that the presence of HAI in CHD-PAH is closely associated with poor prognosis. In the present study, we also explore the difference of clinical characteristics in CHD-PAH. In our opinion, more attention should be paid to the problem of central venous catheterization. Aiming at its clinical features, effective measures should be taken to strengthen the prevention and control of CLABSI. In order to minimize the occurrence of HAI, the following measures were recommended for reference according to our experience: minimize the indwelling time, strengthen the training of clinical medical staff, be proficient in catheterization and maintenance techniques, strictly observe hand hygiene and aseptic technical operation, adopt the maximum aseptic barrier, regularly evaluate the necessity of catheter indwelling, remove any unnecessary vascular catheters as early as possible, and so on.

Some limitations still existed in the present study. First, the effect of HAI on the clinical outcomes of patients with CHD-PAH in PICU was not considered. Second, more factors can be future included to explore the risk factors of HAI in patients with CHD-PAH, as well as the effective measures to prevent HAI. Third, since the pandemic of COVID-19, both the management strategies and clinical characteristics of HAI have changed a lot (20), which was neglected in our research.

## Conclusion

In summary, HAIs are easy to occur in PICU, especially in patients with CHD-PAH. G-bacteria is the predominant pathogen, which requires reasonable selection of antibiotics and strict aseptic operation. To reduce unnecessary invasive procedures will be helpful for the decreased incidence of postoperative HAI in children with CHD-PAH. Our study comprehensively describes the surveillance data of HAI in PICU from a tertiary hospital in western China, and firstly focuses on the subpopulation with CHD-PAH, which provides evidence for clinical prevention and control.

## Data Availability

The raw data supporting the conclusions of this article will be made available by the authors, without undue reservation.

## References

[1] Murni IK Duke T Kinney S Daley AJ Wirawan MT Soenarto Y. Risk factors for healthcare-associated infection among children in a low-and middle-income country. BMC Infect Dis. (2022) 22(1):406. 10.1186/s12879-022-07387-2 35473658 PMC9040216

[2] Spicer KB Green J Dhada B. Hospital-acquired infections in paediatric medical wards at a tertiary hospital in KwaZulu-natal, South Africa. Paediatr Int Child Health. (2018) 38(1):53–9. 10.1080/20469047.2017.1299897 28300495

[3] Beshay S Sahay S Humbert M. Evaluation and management of pulmonary arterial hypertension. Respir Med. (2020) 171:106099. 10.1016/j.rmed.2020.106099 32829182

[4] Pascall E Tulloh RM. Pulmonary hypertension in congenital heart disease. Future Cardiol. (2018) 14(4):343–53. 10.2217/fca-2017-0065 29792339 PMC6136120

[5] Schwartz SS Madsen N Laursen HB Hirsch R Olsen MS. Incidence and mortality of adults with pulmonary hypertension and congenital heart disease. Am J Cardiol. (2018) 121(12):1610–6. 10.1016/j.amjcard.2018.02.051 29655882

[6] Zhang J Yuan Y Li P Wang T Gao J Yao J Postoperative nosocomial infections among children with congenital heart disease. Pak J Med Sci. (2014) 30(3):554–7. 10.12669/pjms.303.4648 24948978 PMC4048505

[7] Galie N Humbert M Vachiery JL Gibbs S Lang I Torbicki A 2015 ESC/ERS guidelines for the diagnosis and treatment of pulmonary hypertension: the joint task force for the diagnosis and treatment of pulmonary hypertension of the European society of cardiology (ESC) and the European respiratory society (ERS): endorsed by: association for European paediatric and congenital cardiology (AEPC), international society for heart and lung transplantation (ISHLT). Eur Heart J. (2016) 37(1):67–119. 10.1093/eurheartj/ehv317 26320113

[8] Ministry of Health of the People’s Republic of China. Diagnostic criteria for nosocomial infections. Chin Med J. (2001) 81:314–20.

[9] Hanley S Odeniyi F Feemster K Coffin SE Sammons JS. Epidemiology and risk factors for healthcare-associated viral infections in children. J Pediatric Infect Dis Soc. (2021) 10(10):941–50. 10.1093/jpids/piab015 34313773

[10] Leoncio JM Almeida VF Ferrari RAP Capobiango JD Kerbauy G Tacla M. Impact of healthcare-associated infections on the hospitalization costs of children. Rev Esc Enferm USP. (2019) 53:e03486. 10.1590/s1980-220x2018016303486 31433016

[11] Akinkugbe O Cooke FJ Pathan N. Healthcare-associated bacterial infections in the paediatric ICU. JAC Antimicrob Resist. (2020) 2(3):dlaa066. 10.1093/jacamr/dlaa066 34223023 PMC8210264

[12] Murni IK Duke T Kinney S Daley AJ Laksanawati IS Nurnaningsih Multifaceted interventions for healthcare-associated infections and rational use of antibiotics in a low-to-middle-income country: can they be sustained? PLoS One. (2020) 15(6):e0234233. 10.1371/journal.pone.0234233 32544154 PMC7297356

[13] Arif S Sadeeqa S Saleem Z Latif S Sharif M. The burden of healthcare-associated infections among pediatrics: a repeated point prevalence survey from Pakistan. Hosp Pract. (2021) 49(1):34–40. 10.1080/21548331.2020.1826783 32990488

[14] Hatachi T Tachibana K Takeuchi M. Incidences and influences of device-associated healthcare-associated infections in a pediatric intensive care unit in Japan: a retrospective surveillance study. J Intensive Care. (2015) 3:44. 10.1186/s40560-015-0111-6 26509039 PMC4621933

[15] Atici S Soysal A Kepenekli Kadayifci E Karaaslan A Akkoc G Yakut N Healthcare-associated infections in a newly opened pediatric intensive care unit in Turkey: results of four-year surveillance. J Infect Dev Ctries. (2016) 10(3):254–9. 10.3855/jidc.7517 27031457

[16] Constantine A Dimopoulos K Opotowsky AR. Congenital heart disease and pulmonary hypertension. Cardiol Clin. (2020) 38(3):445–56. 10.1016/j.ccl.2020.04.008 32622496

[17] Kimura D McNamara IF Wang J Fowke JH West AN Philip R. Pulmonary hypertension during respiratory syncytial virus bronchiolitis: a risk factor for severity of illness. Cardiol Young. (2019) 29(5):615–9. 10.1017/S1047951119000313 31104634

[18] Ren C Wu C Pan Z Wang Q Li Y. Pulmonary infection after cardiopulmonary bypass surgery in children: a risk estimation model in China. J Cardiothorac Surg. (2021) 16(1):71. 10.1186/s13019-021-01450-w 33827623 PMC8025064

[19] Kimura D Saravia J Jaligama S McNamara I Vu LD Sullivan RD New mouse model of pulmonary hypertension induced by respiratory syncytial virus bronchiolitis. Am J Physiol Heart Circ Physiol. (2018) 315(3):H581–9. 10.1152/ajpheart.00627.2017 29906223 PMC6172644

[20] Parriott AM Kazerouni NN Haridass V Barahmani N Palmer LG Winston DT Healthcare-associated infection reporting completeness and quality during the coronavirus disease 2019 (COVID-19) pandemic in California hospitals. Infect Control Hosp Epidemiol. (2022):1–3. 10.1017/ice.2022.247 PMC9588412 36226809

